# Elastic stable intramedullary nailing alone for unicameral bone cysts of the humerus in children: a mid-term follow-up study

**DOI:** 10.3389/fped.2026.1760767

**Published:** 2026-01-29

**Authors:** Shoushen Liu, Fujiang Li, Yanzhou Wang, Mingang Zhang, Tianyou Li

**Affiliations:** 1 Department of Adolescent Sports Medicine, Qingdao Municipal Hospital, Qingdao, China; 2 Department of Pediatric Orthopedics, Shandong Provincial Hospital Affiliated to Shandong First Medical University, Jinan, China

**Keywords:** Capanna classification, children, elastic stable intramedullary nail, humerus, unicameral bone cyst

## Abstract

**Introduction:**

Unicameral bone cyst (UBC) is a common disease in children. There are several different treatment modalities for this condition. Elastic stable intramedullary nail (ESIN) insertion alone was a minimally invasive method that allows for cyst drainage and fracture prevention; however, the treatment outcome remains unclear and inadequately classified. We aimed to evaluate the outcomes of pediatric humeral UBC treated only with ESIN insertion.

**Methods:**

This was a retrospective case series study. Data records included patient demographics, fracture classification, cyst size, treatment strategy, and complications. The Capanna classification was used to determine the outcomes. The potential influential factors for complete healing were analyzed.

**Results:**

A total of 22 patients with an average age at surgery of 7.2 years were included. The average follow-up was 5.0 years (range 2.1–12 years). There were 3 (13.64%) type I, 8 (36.36%) type II, 3 (13.64%) type III, 8 (36.36%) type IV. 2163;. The complete healing rate (type I) and effective rate (type I, II) were 13.64% and 50%, respectively. No factors significantly influenced complete healing. At the last follow-up visit, none of the patients experienced pain or refracture, and their shoulder motion was unlimited.

**Conclusion:**

In our study, the use of ESIN insertion alone to treat humeral UBC, on the basis of the Capanna classification, resulted in a complete healing rate of 13.64% and an overall effective rate of 50%.

## 1 Introduction

Unicameral bone cyst (UBC), also known as simple bone cyst (SBC), was a common tumor-like lesion in children, and accounted for approximately 3% of all primary bone tumors (1). It predominantly occurred at the metaphysis of long bones, most frequently at the proximal humerus and proximal femur, followed by the tibia, fibula, radius, and ulna. Involvement of the pelvis, calcaneus, mandible, and other sites was extremely rare. The onset of UBC was insidious, and there was no pain or limited joint movement in the early stage; most of them were treated for pathological fractures after trauma.

Since UBC was first described by Rudolph Virchow et al. in 1876 (2), its etiology and pathogenesis have not been fully elucidated, which may be related to trauma, infection, sinus vascular block, bone resorption, and venous obstruction. However, some studies have suggested that the main mechanism involves increased pressure within the cyst due to the obstruction of venous return (3). The therapeutic methods, including conservative treatment, curettage, bone-grafting (autografting, allografting, demineralized bone matrix, and artificial bone substitution), percutaneous steroid or autologous bone marrow injection, and decompression and drainage with cannulated screws or elastic stable intramedullary nail (ESIN) (2, 4, 5), remain controversial.

ESIN has been widely used since it was first applied to UBC by Roposch et al. (6); nevertheless, treatment of UBC only with ESIN has been rarely reported until now. When ESIN was applied alone, it was simple to operate and had the advantage of being minimally invasive, which could achieve the clinical goals of cyst drainage and fracture prevention. However, the efficacy reported by different studies varied greatly, with a healing rate of 32%–100% (5–9). However, in our experience, the healing rate was lower than reported. This study retrospectively analyzed cases of pediatric humeral UBC treated with ESIN alone, aiming to provide a reference for clinicians.

## 2 Materials and methods

### 2.1 Inclusion and exclusion criteria

Inclusion criteria: (1) patients who were younger than 18 years; (2) patients with humeral UBC confirmed by x-ray radiographs, no matter whether with pathological fractures or not; (3) patients treated only with ESIN; and (4) patients with complete medical records and radiographic data.

Exclusion criteria: (1) patients who were followed up for less than 24 months; and (2) patients who were lost to follow-up.

### 2.2 Patient data and grouping

The general data of 25 children with humeral UBC who were treated only with ESIN in Shandong Provincial Hospital from June 2009 to July 2021 were retrospectively analyzed.

In accordance with the Capanna classification criteria (10), the final follow-up outcomes were divided into the following four categories: (Ⅰ) healed, (Ⅱ) healed with residual, (Ⅲ) recurrence, and (Ⅳ) no response.

In order to study the influential factors for complete healing, the children were divided into a completely healed group (Group A) and an incompletely healed group (Group B) according to whether they could be classified as Capanna type Ⅰ, and potential factors were measured and compared between the two groups.

This study was approved by the Biomedical Research Ethics Committee of Shandong Provincial Hospital (No. SWYX2022-589).

### 2.3 Diagnostic methods

A confirmed diagnosis of humeral UBC was based on detailed history collection, physical examination, and typical radiographic signs (8, 9, 11–13). All the imaging diagnoses were made by a senior consultant radiologist in our institution. To ensure accuracy, each patient was diagnosed clinically by two senior pediatric orthopedists (the second author and the corresponding author).

### 2.4 Surgical procedures

The patient was placed in the supine position, and the affected arm was sterilized after general anesthesia. A 2 cm longitudinal incision was made around the lateral epicondyle of the humerus. The first ESIN was prebent into a “C” shape and rotated retrogradely to the lesion under the guidance of C-arm fluoroscopy. When the displacement of the pathological fracture was obvious, the assistants cooperated with traction and reduction of the fracture ends. After satisfactory reduction, the ESIN was inserted toward the proximal epiphyseal plate of the humerus. The second ESIN was inserted through the lateral or medial incision: (1) Lateral incision: The second ESIN was prebent into an “S” shape, and the entry point was located 1 cm to 2 cm from the first; (2) Medial incision: A 2 cm incision was made around the medial epicondyle of the distal humerus and the ESIN was retrogradely inserted in the same way. The two ESINs were placed into a “C & S” or double “C” configuration and occupied two-thirds of the diameter of the medullary cavity (6). C-arm fluoroscopy was used to confirm the location of the ESIN in the medullary cavity and to ensure that the nail tip did not injure the epiphysis. The incisions were sutured in layers and wrapped in a sterile dressing.

### 2.5 Measurement data

**Figure 1:**
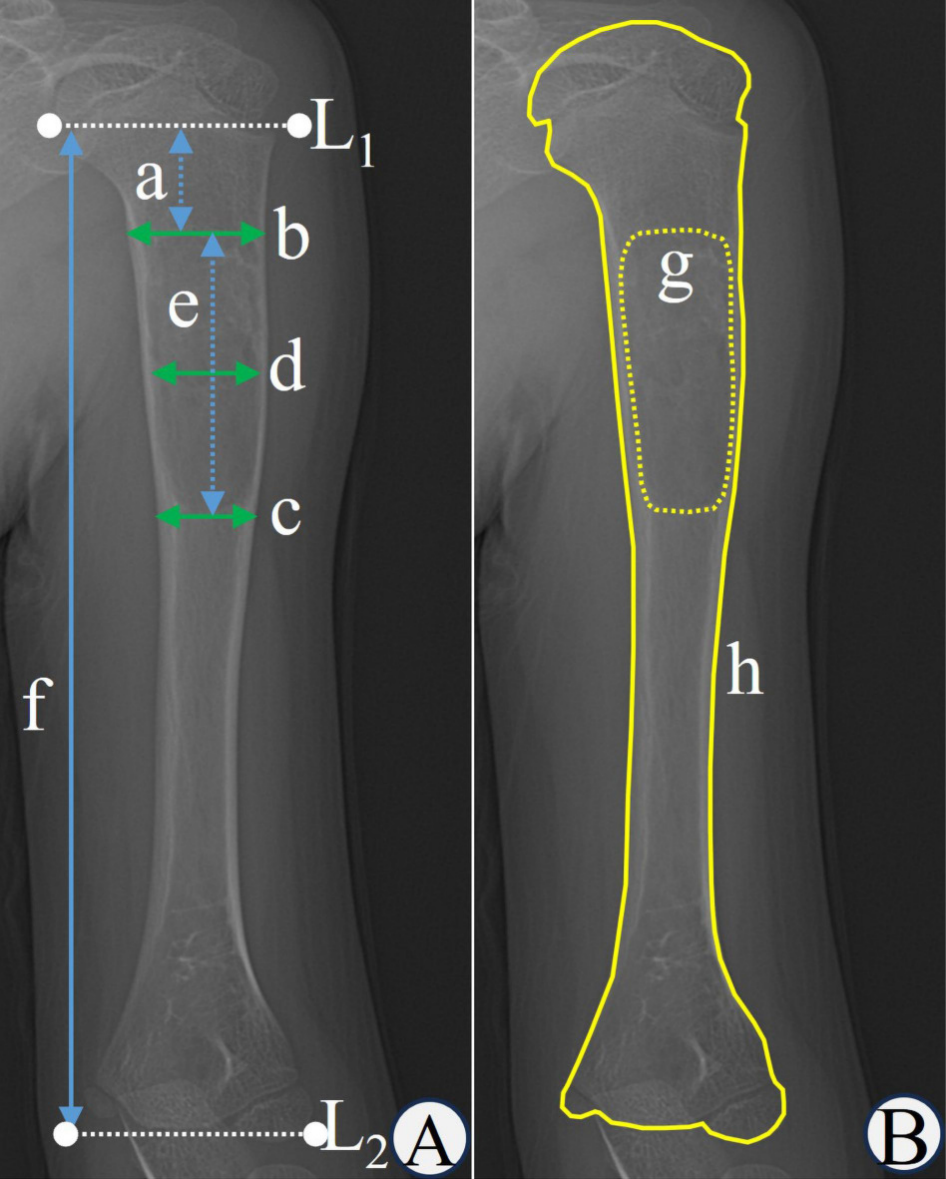
**(a)** the distance from the proximal cyst edge to the physis; **(b)** the humeral width adjacent to the proximal edge of the cyst; **(c)** the humeral width adjacent to the distal edge of the cyst; **(d)** the cyst width; **(e)** the cyst length; **(f)** the humeral length; **(g)** the cyst area; **(h)** the humeral area; (L1) the junction of the proximal humeral metaphysis and the epiphysis; (L2) the junction of the distal humeral metaphysis and the epiphysis.

On the anteroposterior radiographs, auxiliary lines L1 and L2 were drawn at the transition between the proximal and distal humeral metaphysis and the epiphysis, respectively. Photoshop 2021 (Adobe, USA) was used to measure the distance from the proximal cyst edge to the physis (a), the humeral width adjacent to the proximal edge of the cyst (b), the humeral width adjacent to the distal edge of the cyst (c), the cyst width (d), the cyst length (e) and the humeral length (f). Image J 1.53a (NIH, USA) was used to measure the cyst area (g) and the humeral area (h) on the anteroposterior radiograph. In this study, the humeral width was defined as the average of the sum of the humeral width adjacent to the proximal and distal edge of the cyst, the humeral length was defined as the distance between the L1 and L2, and the humeral area was defined as the visible bony area (Figure 1B) and did not include the unvisualized proximal and distal humeral cartilage. Two pediatric orthopedic surgeons measured the data using the above software, and the average was calculated. When the two data sets differed significantly, a third surgeon performed the measurements again, and the average was calculated. The measurement method was shown in Figure 1.

### 2.6 Fracture classification methods

**Figure 2:**
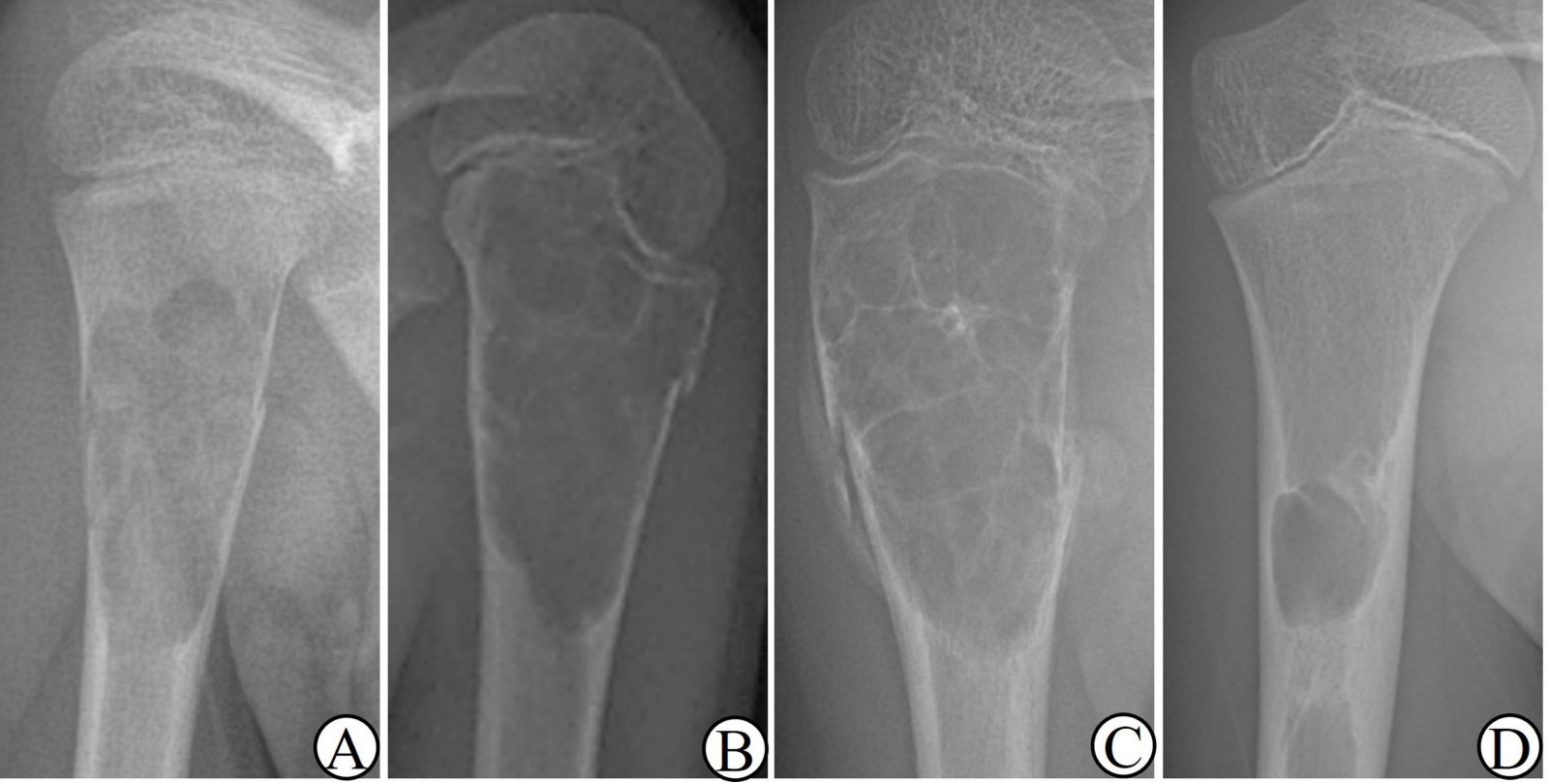
**(A)** complete fracture; **(B)** incomplete fracture; **(C)** old fracture; **(D)** non-fracture.

According to the radiographs at presentation, patients were first divided according to whether they had a fracture. Cases with fractures were classified by the fracture time into fresh and old fractures (≥3 weeks). For fresh fractures, they were subdivided into complete (total break of the bone cortex or obvious displacement) and incomplete fractures. The classification method was shown in Figure 2.

### 2.7 Statistical methods

SPSS 27.0 software (IBM Corp., USA) was used for the statistical analysis. Fisher's exact test was used to compare the differences in sex, side, surgical history, fracture or not, fracture time, complete fracture or not, and the number of ESIN between the two groups. Independent sample *t-*test was used to compare age, the ratio of the cyst length to humeral length, the cyst width to the humeral width, the cyst length to cyst width, and the cyst area to humeral area. The rank sum test was used to compare the ratio of the distance from the proximal cyst edge to the physis to the humeral length. *P* < 0.05 was considered statistically significant.

## 3 Results

### 3.1 General data

Twenty-two patients were enrolled in the study, including 20 boys and 2 girls, with an average age of 7.2 years (range 3–13 y) and an average follow-up of 5.0 years (range 2.1–12 y). There were 9 cases on the left side and 13 cases on the right side. Among the 22 children, 3 had nonfracture, and the remaining 19 cases included 10 old fractures and 9 fresh fractures. The fresh fractures were further divided into 7 complete fractures and 2 incomplete fractures. The general data of the patients were shown in Table 1.

**Table 1:** General data of 22 patients with UBC in the humerus.

N	Y	Sex	S	Surgical history	Fracture or not	Number of ESIN	Follow-up period (years)	Capanna classification	Secondary treatment
1	10	M	R	No	Yes	2	11.1	II	-
2	6	M	L	Yes	Yes	1	10.4	IV	-
3	7	M	L	No	Yes	2	12.0	III	Curettage and bone-grafting
4	4	M	R	No	Yes	1	7.3	IV	-
5	4	M	R	No	No	2	7.2	II	Bone marrow injection + ESINs
6	6	M	R	Yes	Yes	1	6.2	II	-
7	13	F	R	No	Yes	2	6.1	III	-
8	3	M	R	Yes	Yes	2	5.0	II	-
9	8	M	R	No	Yes	2	5.0	I	-
10	7	M	L	No	Yes	2	4.8	IV	Curettage and bone-grafting + ESINs
11	9	M	R	No	Yes	2	4.6	I	-
12	3	M	L	No	Yes	2	4.0	II	ESINs replacement
13	5	M	R	No	Yes	2	3.9	IV	-
14	6	M	L	No	No	1	3.3	I	-
15	11	M	R	No	Yes	2	3.2	IV	ESINs replacement
16	7	M	L	No	Yes	1	3.0	II	ESINs replacement
17	5	F	L	No	Yes	2	2.7	IV	-
18	9	M	R	No	Yes	2	2.5	II	-
19	7	M	R	No	Yes	2	2.4	IV	-
20	12	M	L	No	Yes	2	2.2	III	-
21	11	M	R	No	No	1	2.2	II	-
22	6	M	L	No	Yes	1	2.1	IV	Curettage and bone-grafting + ESINs

N, Number; Y, Years; M, Male; F, Female; S, Side; L, Left; R, Right; “-“ means no secondary treatment was performed.

### 3.2 Treatment outcome

**Figure 3:**
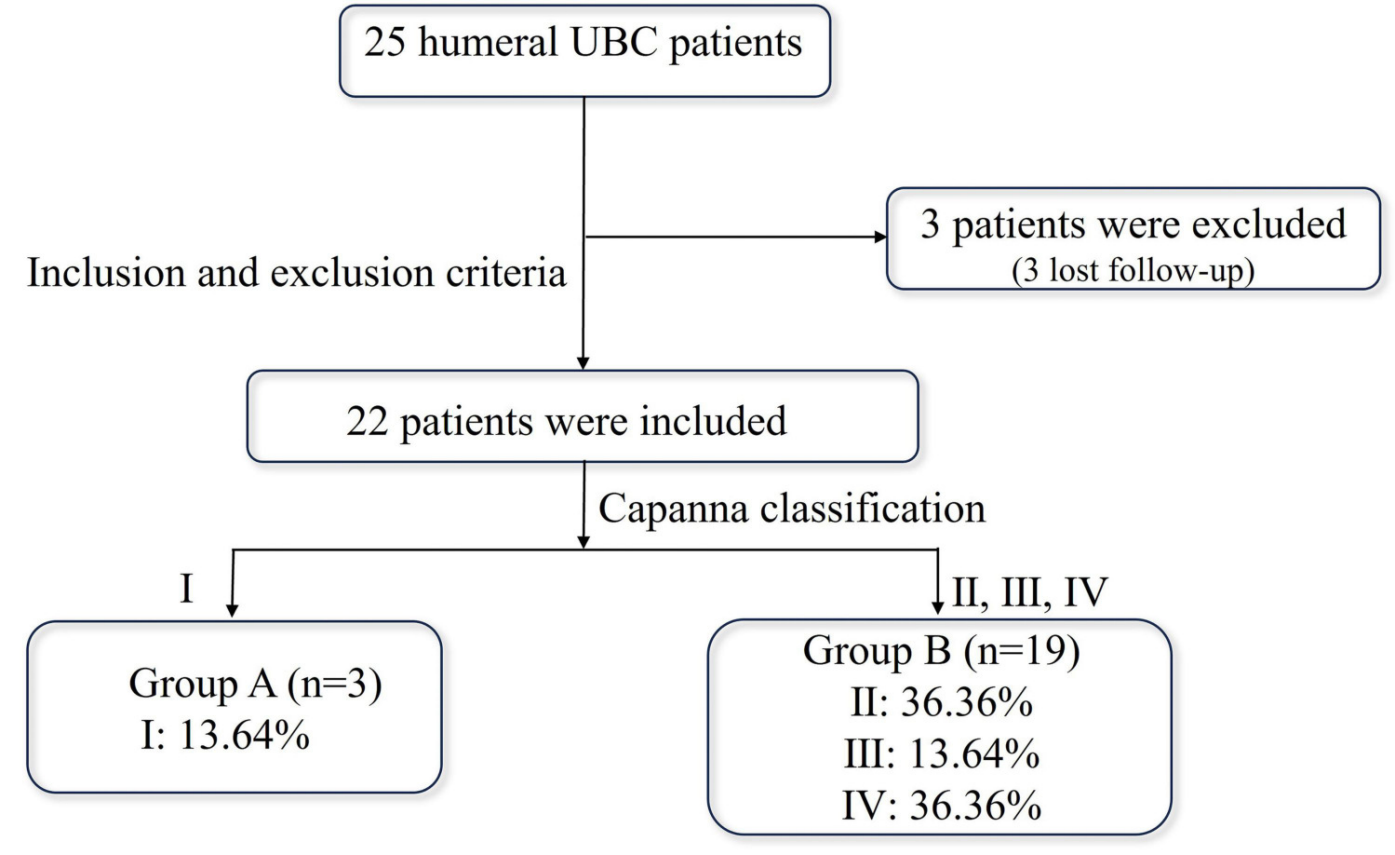
The result of grouping.

**Figure 4:**
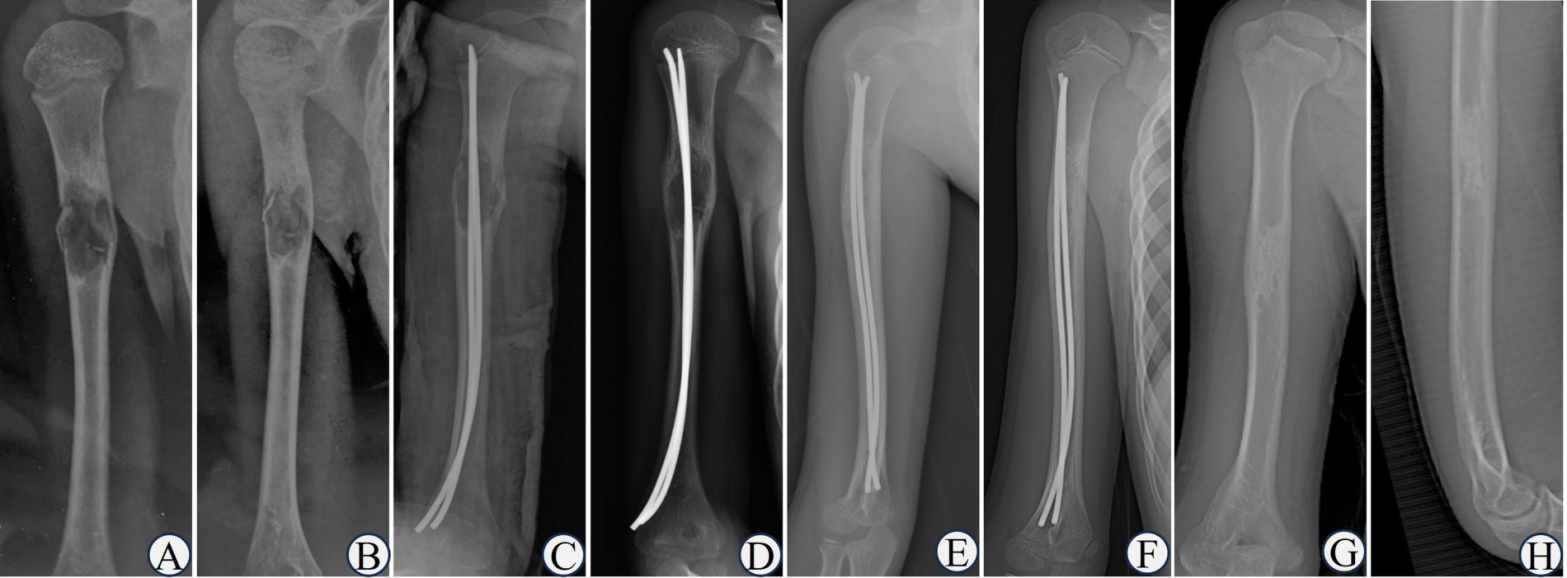
Typical case of capanna type I. 2160;. Boy, 8-year-old, Case NO 9, UBC of right humerus. **(A,B)** the humeral UBC sustained pathological fractures after trauma; **(C)** double ESINs for decompression and drainage; **(D)** 1 month after the operation, cortical bone thickened and the cyst area decreased; **(E)** the osteolytic lesions disappeared after 6 months; **(F)** healed after 1 year; **(G,H)** completely healed after 5 years.

**Figure 5:**
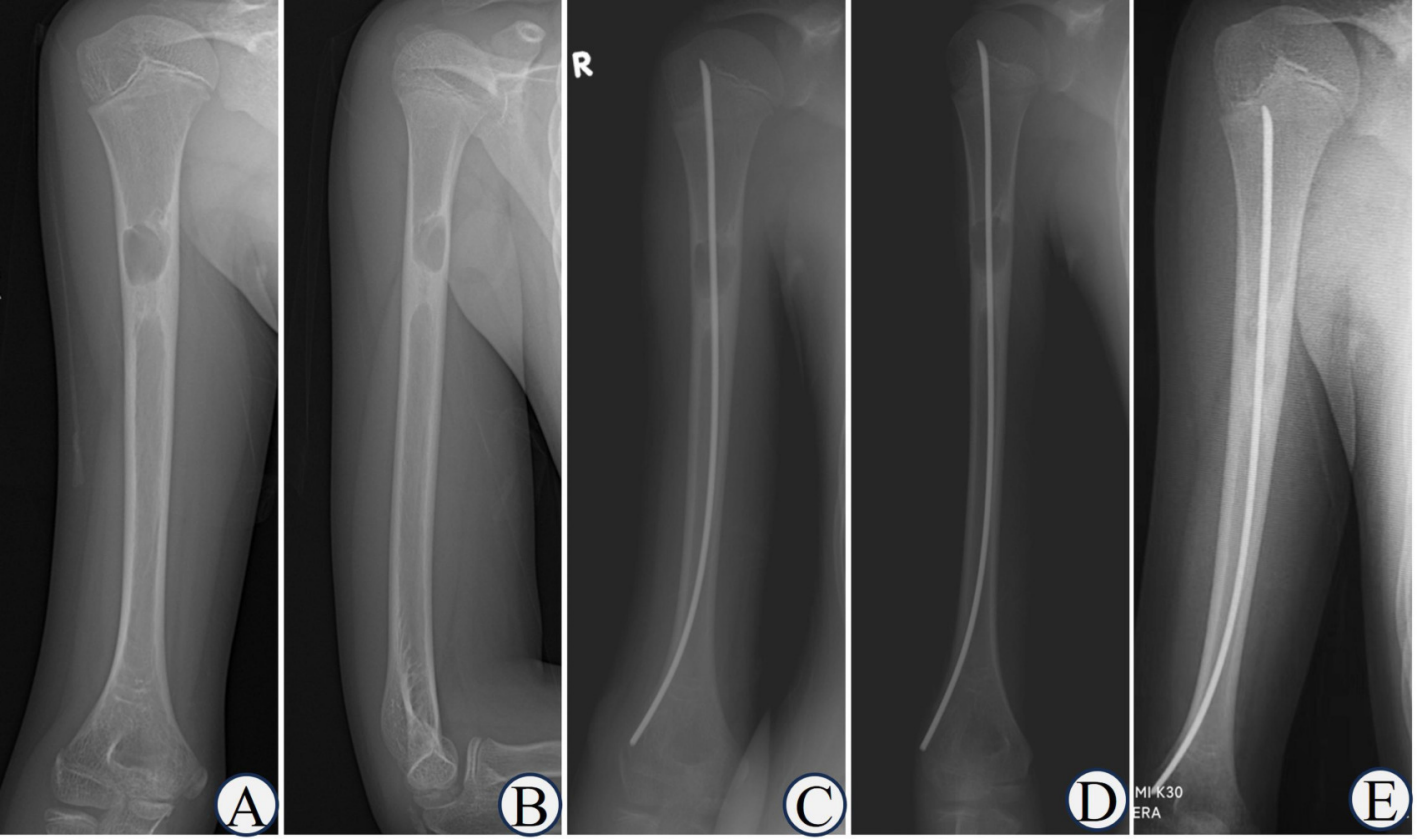
Typical case of capanna type II. 2161;. Boy, 11-year-old, Case NO 21, UBC of right humerus. **(A,B)** humeral UBC without pathological fractures; **(C,D)** single ESIN for decompression and drainage; **(E)** the residual lesion was obvious after 2 years.

**Figure 6:**
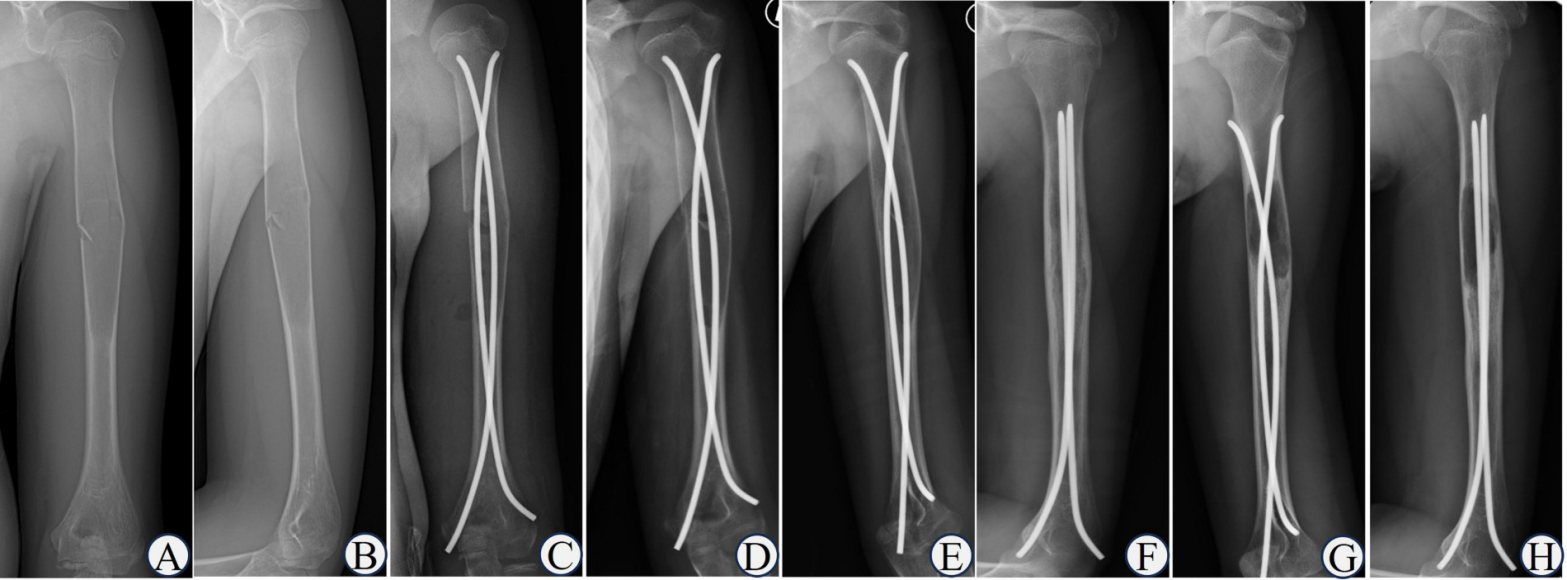
Typical case of capanna type Ⅲ. Boy, 12-year-old, Case NO 20, UBC of left humerus. **(A,B)** humeral UBC with pathological fractures; **(C)** double ESINs for decompression and drainage; **(D)** the fracture line was blurred after 2 weeks; **(E)** the fracture healed after 2 months; **(F)** the cyst healed after 1 year; **(G,H)** the cyst recurred after 2 years.

**Figure 7:**
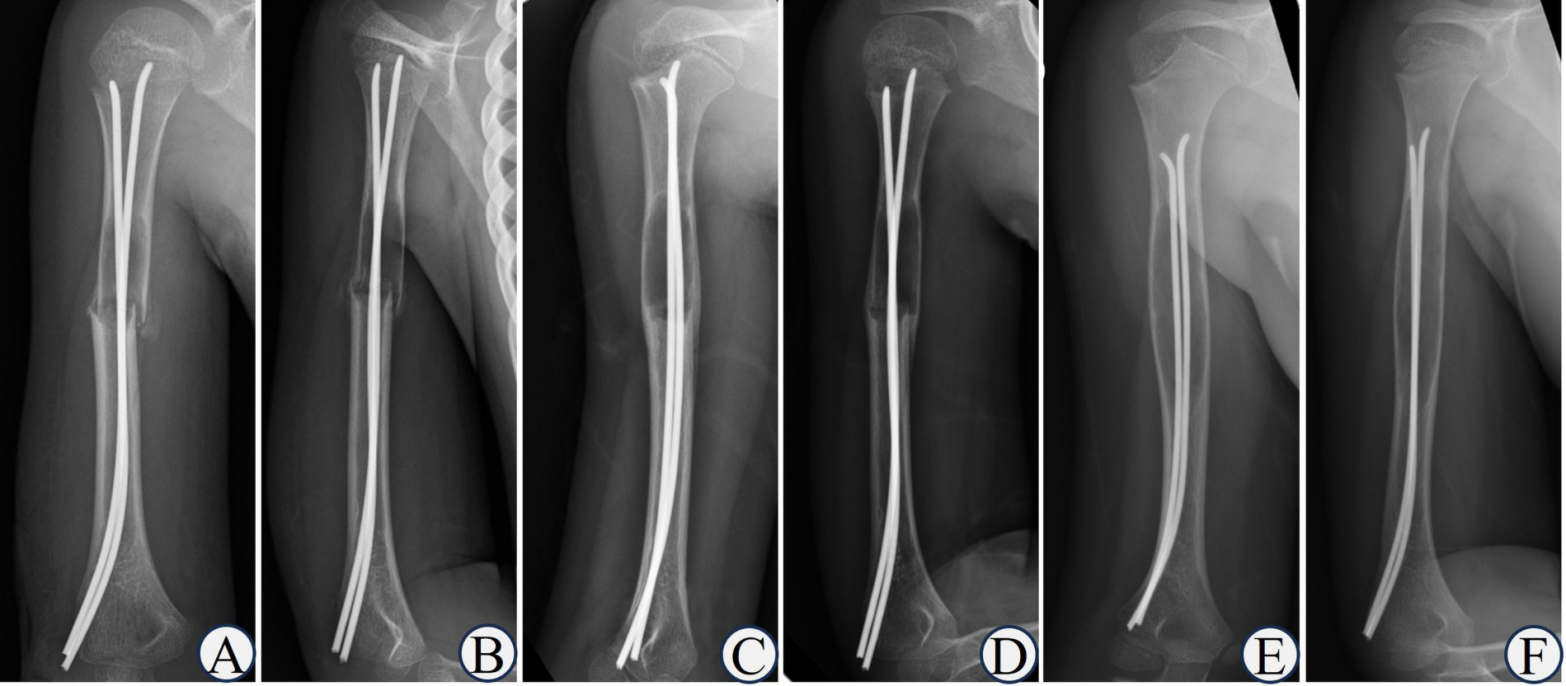
Typical case of capanna type Ⅳ. Boy, 7-year-old, Case NO 19, UBC of right humerus. **(A,B)** humeral UBC sustained pathological fractures; **(C,D)** the callus was abundant after 6 weeks; **(E,F)** the cyst size did not decrease or even expand after 2.5 years.

According to the Capanna grading criteria, there were 3 (13.64%) cases of type Ⅰ, forming Group A; and 8 (36.36%) cases of type Ⅱ, 3 (13.64%) cases of type Ⅲ, 8 (36.36%) cases of type Ⅳ, forming Group B. Both types Ⅰ and Ⅱ responded to treatment, with the complete healing rate (type Ⅰ) being 13.64%, and the effective rate (type Ⅰ and Ⅱ) was 50%. The flowchart of the grouping was shown in Figure 3. The typical cases were shown in Figures 4–7.

### 3.3 Analysis of influential factors

Between the two groups, univariate analysis of sex, age, side, surgical history, fracture or not, fracture time, complete fracture or not, the number of ESIN, and the ratios of the distance from the proximal cyst edge to the physis to the humeral length, cyst length to humeral length, cyst width to humeral width, cyst length to cyst width, and cyst area to humeral area showed no statistically significant differences (*P* > 0.05, Tables 2–4).

**Table 2:** The results of the univariate analysis between the two groups (*n* = 22).

Variable	Group A (*n* = 3)	Group B (*n* = 19)	*t*/*z*-value	*P*-value
Sex				
male	3 (100.00%)	17 (89.47%)		1.000
female	0 (0.00%)	2 (10.53%)		
Age	7.67 ± 1.53	7.16 ± 3.04	−0.280	0.782
Side				
left	1 (33.33%)	8 (42.11%)		1.000
right	2 (66.67%)	11 (57.89%)		
Surgical history				
yes	0 (0.00%)	3 (15.79%)		1.000
no	3 (100.00%)	16 (84.21%)		
Fracture or not				
yes	2 (66.67%)	17 (89.47%)		0.371
no	1 (33.33%)	2 (10.53%)		
Number of ESINs				
1	1 (33.33%)	6 (31.58%)		1.000
2	2 (66.67%)	13 (68.42%)		
Cyst length/humeral length	0.15 ± 0.04	0.29 ± 0.12	1.876	0.075
Cyst width/humeral width	0.91 ± 0.07	0.94 ± 0.15	0.288	0.777
Cyst length/cyst width	1.73 ± 0.35	2.68 ± 0.95	1.684	0.108
Cyst area/humeral area	0.13 ± 0.02	0.23 ± 0.12	1.387	0.181
The distance from the proximal cyst edge to the physis/the humeral length^a^	0.18 (0.07, -)	0.11 (0, 0.20)	−0.844	0.438

a "*" indicates that the variable does not follow the normal distribution.

“-” indicates a blank space.

**Table 3:** The result of the chi-square test for fracture time between the two groups (*n* = 19).

Variable	Group A (*n* = 2)	Group B (*n* = 17)	*P*-value
Fracture time			
<3 weeks	2 (100.00%)	7 (41.18%)	0.211
≥3 weeks	0 (0.00%)	10 (58.82%)	

*: 3 cases of non-fracture were excluded.

**Table 4:** The result of the chi-square test for complete fracture or not between the two groups (*n* = 9).

Variable	Group A (*n* = 2)	Group B (*n* = 7)	*P*-value
Complete fracture or not			
yes	2 (100.00%)	5 (71.43%)	1.000
no	0 (0.00%)	2 (28.57%)	

*: 3 cases of non-fracture and 10 cases of old fracture were excluded.

### 3.4 Complications

At the last follow-up visit, all the patients had no pain at the lesion site, and the motion range of the shoulder joint was satisfactory enough for physical activities. There were no complications such as infection, refracture, nerve injury, physeal arrest, or upper limb length discrepancy.

### 3.5 Secondary treatment

The classification and clinical assessment were made before the additional intervention if used. For additional procedures, 2 type Ⅱ patients and 1 type Ⅳ patient underwent ESIN replacement, 1 type Ⅱ patient received bone marrow injection and ESIN change, 1 type Ⅲ patient was treated with curettage and bone grafting, and 2 type Ⅳ patients underwent curettage, bone grafting, and ESIN change.

## 4 Discussion

UBC has certain self-healing properties, but often requires active surgical intervention due to pathological fractures (3, 13). The treatment purpose was to promote the healing of cysts, immobilize, and prevent pathological fractures. Given that the etiology and pathogenesis were still unclear, there has been no consensus on the optimal surgical technique and time for UBC so far.

ESIN insertion has several advantages: (1) The operation is a minimally invasive procedure with low intraoperative bleeding and a short hospital stay. (2) The nail tip can reduce cystic fluid generation by destroying the inner wall of the cyst so that the cavity can form an osteogenic microenvironment to promote ossification (14). (3) ESIN provides continuous decompression and drainage by penetrating the cystic cavity and medullary cavity; meanwhile, mesenchymal stem cells in bone marrow can enter the cyst, promoting cyst healing by its high osteoinductive and osteogenic capacity. (4) ESIN can immobilize existing pathological fractures and effectively prevent refractures.

Currently, the tools used to evaluate the efficacy of UBC include the Neer classification (15), Capanna classification (10), and Chang classification (16). Among the aforementioned classifications, the Capanna classification was selected for this study due to its most extensive application. In previous literature, patients classified as Capanna Ⅰ and Ⅱ were regarded as successful treatments. However, this study aimed to assess what kind of lesions could be completely healed; thus, only type Ⅰ was seen as complete healing, and all the other types were considered incomplete healing. Interestingly, it was worth noting that among patients of type II, there were some special cases where the residual cyst range was smaller than that before the operation, but it was still significantly extensive, even exceeding half of the diameter of the humerus. To better guide clinical treatment, our future research will focus on these special cases, refine the classification, and conduct external validation.

In our cohort, the complete healing rate of humeral UBC treated only with ESIN was 13.64%, and the effective rate was 50%, which differed from the results reported in previous literature (6–9, 17–19). Mavčič et al. (19) compared three surgical treatment options for humeral UBC, and the complete healing rate of the ESIN group was 32%. Roposch et al. (6) used ESIN to treat 32 cases of UBC; 93.75% of the children presented with pathological fractures, and the complete healing rate was 43.75%. Masquijo et al. (8) analyzed 48 cases of long bone UBC treated with continuous decompression and drainage with ESIN. After 9.8 years of follow-up, the complete healing rate was 54.17% (26/48), and the authors reported no significant difference in the healing rate between humeral and femoral lesions. Knorr et al. (9) used ESIN to treat 15 cases of UBC of the proximal humerus in children with pathological fractures and reported that 66.67% of the children healed completely within 3 years after the operation.

To analyse, the reasons for the lower complete healing rate may include the following: (1) The general data of patients in our group were not completely consistent with those in the aforementioned literature, including ethnicity, male-to-female ratio, lesion sites, initial symptoms, and proportion of pathological fractures, etc. (2) The subjectivity of evaluators may introduce error into outcomes. As illustrated in the first figure from the study reported by de Sanctis et al. (11), residual lesions persisted at the proximal femur during final follow-up, yet this case was classified as type I. This discrepancy may explain the higher rates of complete healing reported in the literature. Therefore, we recommend that efficacy evaluations be conducted by at least two investigators. In cases of conflicting results, further guidance should be sought from a senior-level specialist. (3) The healing classification might change during follow-up. For children who showed no obvious residual lesion on radiographs and looked fully healed, the cyst might recur, such as the patient in Figure 6. In this case, to ensure the complete healing of the cyst, a CT or MRI should be performed. For children classified as type Ⅱ, it was possible to turn into type Ⅰ during further healing. (4) In the previous literature, all long bone UBC were analyzed together, including the humerus, femur, and tibia. In contrast with the femur, the humerus does not bear weight, leading to differences in treatment selection. This study aimed to evaluate the efficacy of the humerus treated only with ESIN; thus, only humeral UBC were included. The affected site may have a certain influence on the treatment outcomes, which needs further study.

Given the uncertainty of the cyst healing treated with ESIN alone, some authors recommended combined therapy (5, 20–24). Zhang et al. (21) combined ESIN with intralesional steroid injection for the treatment of 18 patients with humeral UBC and achieved ideal results. According to the Capanna classification, the complete healing rate was 77.78%, and the effective rate was as high as 100% (Capanna Ⅰ and Ⅱ). Li et al. (25) used ESIN and bone grafting to treat pathological fractures secondary to femoral UBC and the success rate was 92% (Capanna Ⅰ and Ⅱ). Traub et al*.* (26) reported that the failure rates of treatment with only ESIN, with steroid injection alone, and combined therapy were 50.0%, 36.6%, and 21.4%, respectively, proving that combined treatment was superior to only ESIN or steroid injections.

Some surgeons believe that the healing rate of active UBC was low, and that the location of the cysts was a factor that affects the results (27). The sites of lesions in our group were variable; most were located in the proximal one-third of the humerus, a few were located in the middle one-third of the humerus, and occasionally extensive lesions affected nearly half of the humerus. We did not qualitatively analyze active or latent UBC based on the Neer et al. (15) or Chang et al. (16) classification criteria, but quantitatively evaluated the location of the UBC by calculating the ratio of the distance from the proximal cyst edge to the physis to the humeral length, interestingly, our results revealed that no significant difference in the ratio between the two groups (*P* > 0.05). These findings were consistent with those of Chang et al. (16), who reported that the location of the cyst was not an influential factor in the treatment of humeral and femoral UBC with steroids.

In the literature, two ESINs were mostly used to treat humeral UBC, and the use of one (22, 28–30) and three (31) ESINs has also been reported. The decision on the number of ESIN to use should take into account factors such as the location and extent of the lesion, the thickness of the bone cortex, and whether a pathological fracture was present. In this study, children who received a single ESIN had lesions located in the diaphysis of the humerus, where the lesion size was relatively small and no pathological fracture was present. In cases where the lesion was extensive, located in the metaphysis, the bone cortex was thin, and there was a high risk of pathological fracture or it has already occurred, two ESINs were recommended to improve stabilization.

Owing to the need for secondary anesthesia to remove ESIN, some surgeons may prefer conservative or minimally invasive treatment of humeral UBC with non-displaced fractures, such as steroids, calcium sulfate, or autologous bone marrow injection. Compared with injection therapy, ESIN was superior in enhancing the strength of diseased bone, drainage, and preventing fractures. Although injection therapy was less invasive, it may also require multiple surgeries and anesthesia (30, 32).

Our study had several limitations. First, the diagnosis of most children in this group relied on clinical and radiological features only, without histological confirmation. Second, this study lacked a control group. Third, the number of patients in the group was small, and more patients should be enrolled to verify the efficacy of the treatment.

## 5 Conclusion

In our study, the use of ESIN insertion alone to treat humeral UBC, on the basis of the Capanna classification, resulted in a complete healing rate of 13.64% and an overall effective rate of 50%.

## Data Availability

The raw data supporting the conclusions of this article will be made available by the authors, without undue reservation.
